# ^18^F-Labeled Cyclized α-Melanocyte-Stimulating Hormone Derivatives for Imaging Human Melanoma Xenograft with Positron Emission Tomography

**DOI:** 10.1038/s41598-019-50014-5

**Published:** 2019-09-19

**Authors:** Chengcheng Zhang, Zhengxing Zhang, Helen Merkens, Jutta Zeisler, Nadine Colpo, Navjit Hundal-Jabal, David M. Perrin, Kuo-Shyan Lin, François Bénard

**Affiliations:** 1 Department of Molecular Oncology, BC Cancer, Vancouver, BC Canada; 2 Department of Chemistry, University of British Columbia, Vancouver, BC Canada; 3 Department of Radiology, University of British Columbia, Vancouver, BC Canada

**Keywords:** Cancer imaging, Melanoma

## Abstract

Since metastatic melanoma is deadly, early diagnosis thereof is crucial for managing the disease. We recently developed α-melanocyte-stimulating hormone (αMSH) derivatives, [^68^Ga]Ga-CCZ01048 and [^18^F]CCZ01064, that target the melanocortin 1 receptor (MC1R) for mouse melanoma imaging. In this study, we aim to evaluate [^18^F]CCZ01064 as well as a novel dual-ammoniomethyl-trifluoroborate (AmBF_3_) derivative, [^18^F]CCZ01096, for targeting human melanoma xenograft using μPET imaging. The peptides were synthesized on solid phase using Fmoc chemistry. Radiolabeling was achieved in a one-step ^18^F-^19^F isotope-exchange reaction. μPET imaging and biodistribution studies were performed in NSG mice bearing SK-MEL-1 melanoma xenografts. The MC1R density on the SK-MEL-1 cell line was determined to be 972 ± 154 receptors/cell (n = 4) via saturation assays. Using [^18^F]CCZ01064, moderate tumor uptake (3.05 ± 0.47%ID/g) and image contrast were observed at 2 h post-injection. Molar activity was determined to play a key role. CCZ01096 with two AmBF_3_ motifs showed comparable sub-nanomolar binding affinity to MC1R and much higher molar activity. This resulted in improved tumor uptake (6.46 ± 1.42%ID/g) and image contrast (tumor-to-blood and tumor-to-muscle ratios were 30.6 ± 5.7 and 85.7 ± 11.3, respectively) at 2 h post-injection. [^18^F]CCZ01096 represents a promising αMSH-based μPET imaging agent for human melanoma and warrants further investigation for potential clinical translation.

## Introduction

An estimated 91,270 melanoma cases were diagnosed in the United States in 2018^1^, of which 9,320 will eventually succumb to the disease. The incidence of melanoma has been steadily rising for the past 40 years, and a 46% increase was observed compared to the last decade^2^. The 5-year survival rate is estimated to be only 15–20% for late-stage metastatic melanoma (stage IV)^1^. Even with the advent of new therapies, such as immune checkpoint inhibitors, the 5-year survival rate remains at only 34%^3^. Early diagnosis and accurate staging is thus crucial in managing melanoma.

Positron emission tomography (PET) with 2-[^18^F]fluorodeoxyglucose ([^18^F]FDG) has been successfully used for staging and detecting cutaneous malignant melanoma with high sensitivity (83%) and specificity (85%)^4^ due to the superior sensitivity and spatial resolution compared to other imaging modalities, such as single photon emission computed tomography (SPECT). However, for small metastatic lesions (≤5 mm), only ~23% are detectable by [^18^F]FDG PET^5^. [^18^F]FDG PET imaging also has low sensitivity to detect liver metastases from uveal melanoma^6,7^. This is likely due to the fact that [^18^F]FDG relies on the high glucose metabolic rate typically seen in cancer cells, yet small lesions as well as tumors with low glucose metabolic rates thus elude detection. Moreover, inflammation and infection can also produce high signal intensity with [^18^F]FDG^8^, leading to false positives. Targeting melanoma with a tumor-specific antigen would potentially provide higher specificity and sensitivity.

The most popular molecular targets for μPET imaging of melanoma and their respective ligands in preclinical studies are: (1) melanocortin 1 receptor (MC1R) and α-melanocyte-stimulating hormone (αMSH) peptide derivatives^9,10^; (2) melanin and benzamide-bearing pharmacophores^11,12^; (3) integrin α_v_β_3_ and RGD derivatives^13,14^; and (4) integrin α_4_β_1_ (also known as very late antigen-4, VLA-4) and peptidomimetic ligand LLP2A derivatives^15,16^. The αMSH derivatives represent the most extensively studied radiotracers for melanoma imaging in preclinical animal models owing to higher tumor uptake, faster blood pool clearance, and lower nonspecific accumulation in normal tissues. We recently reviewed the development of αMSH derivatives for melanoma imaging targeting MC1R with μPET and μSPECT, and summarized the biodistribution characteristics of 88 radiolabeled αMSH derivatives evaluated in mice bearing mouse melanoma^17^. Excellent tumor uptake was observed, for instance, 22.8 ± 1.71 percentage of injected dose per gram of tissue (%ID/g) was achieved for [^99m^Tc]Tc-(EDDA)-HYNIC-AocNle-CycMSH_hex_ in mice bearing B16-F1 mouse melanoma at 2 h post-injection (p.i.)^18^. In contrast, when the same radioligand was applied for imaging human M21 melanoma xenograft, only 3.3%ID/g was observed at the same time point^19^. Moreover, in total, only eight αMSH derivatives radiolabeled with SPECT isotopes (^99m^Tc or ^188^Re) were evaluated for imaging human melanoma xenografts, and low tumor uptake (<3.30%ID/g) at 1 or 2 h p.i. was observed^19–22^. μPET imaging of human melanoma with ^18^F in animal models has not yet been successfully developed with αMSH ligands. This is due to the fact that MC1R density in human melanoma generally ranges from a few hundred to a few thousand copies per cell and is 5–20 times less than that in mouse melanoma^21^. The low receptor density presents a significant challenge in developing imaging agents targeting human MC1R.

**Figure 1:**
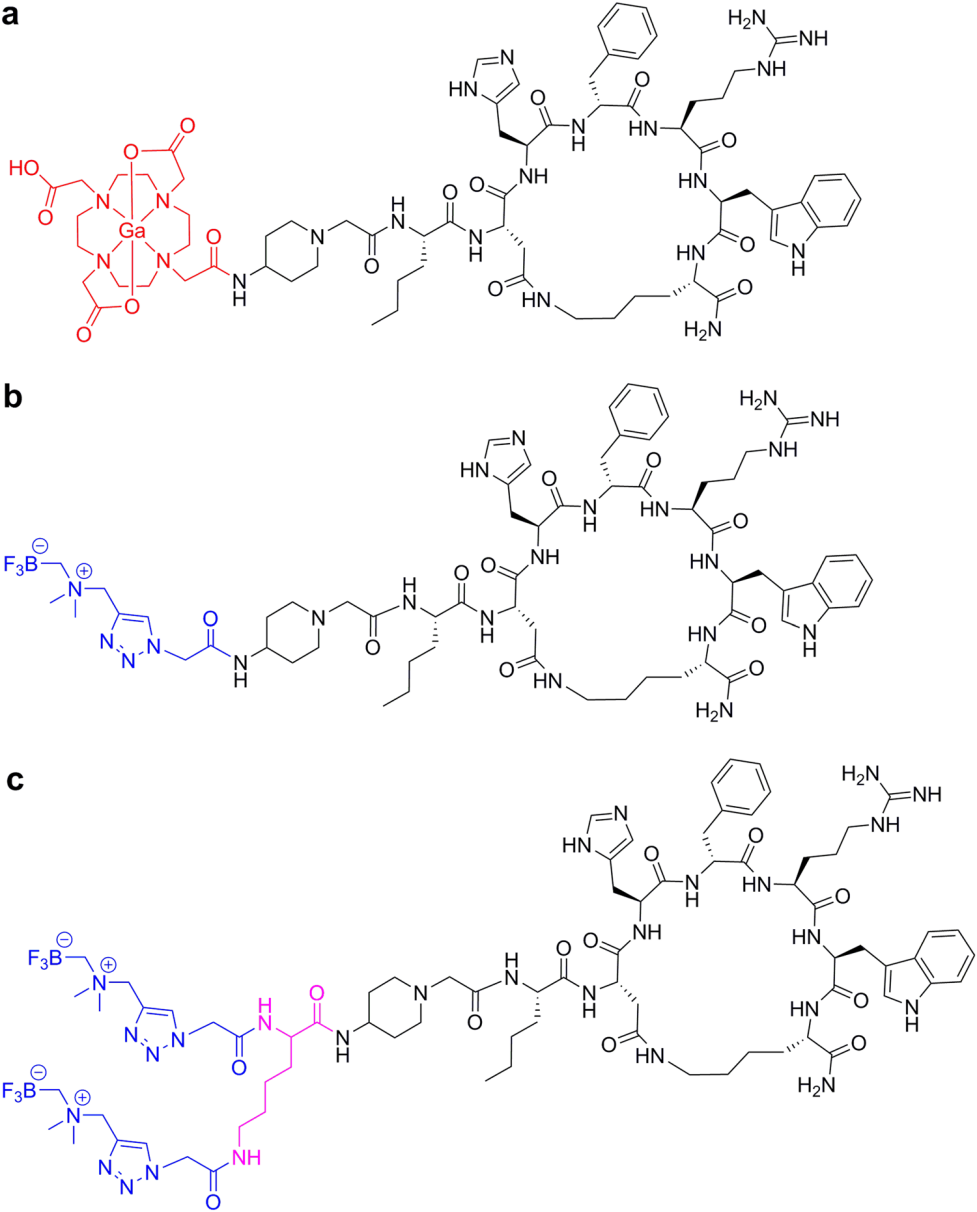
Chemical structures of (**a**) Ga-CCZ01048, (**b**) CCZ01064 and (**c**) a novel dual-ammoniomethyl-trifluoroborate (AmBF_3_) derivative CCZ01096.

We recently developed MC1R-targeting μPET imaging agents, CCZ01048 and CCZ01064 (Fig. 1a,b), based on a cyclized αMSH analogue^9,10^. [^18^F]CCZ01064 and [^68^Ga]Ga-CCZ01048 showed excellent tumor uptake (11.96 ± 2.31 and 21.9 ± 4.6%ID/g at 2 h p.i., respectively) in mice bearing B16-F10 mouse melanoma. The aim of this study was to evaluate the potential of our previously reported CCZ01064 as well as a novel dual-ammoniomethyl-trifluoroborate (AmBF_3_) derivative, CCZ01096 (Fig. 1c), for detecting human melanoma xenografts with μPET in a preclinical mouse model.

## Results

### MC1R expression on SK-MEL-1 human melanoma cell line

**Figure 2:**
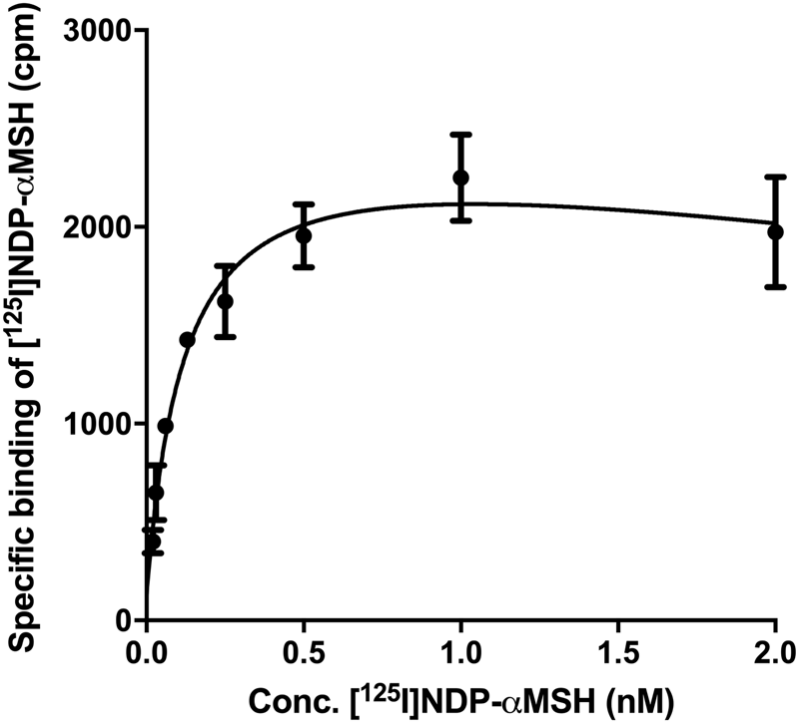
Representative saturation curve using [^125^I]NDP-αMSH on SK-MEL-1 cells.

Among all the human melanoma cell lines with data archived in the Cancer Cell Line Encyclopedia (CCLE)^23^, SK-MEL-1 showed high values in both mRNA expression level and DNA copy number, whereas another human melanoma cell line, MeWo, showed low values in both categories (Supplementary Fig. S1). Western blotting was employed and confirmed that the MC1R protein expression level on SK-MEL-1 cells was higher than MeWo cells (Supplementary Fig. S2). To accurately evaluate the MC1R density on the SK-MEL-1 cell line, receptor saturation assays were performed with increasing concentration of [^125^I]NDP-αMSH (Fig. 2), and the MC1R density was determined to be 972 ± 154 receptors/cell (n = 4).

### Chemistry and MC1R binding affinity

The chemical structures of Ga-CCZ01048, CCZ01064 and CCZ01096 are shown in Fig. 1. Ga-CCZ01048 and CCZ01064 were synthesized as reported previously^9,10^. CCZ01096 was synthesized following similar procedures for the synthesis of CCZ01064 with incorporation of two AmBF_3_ motifs. The chemistry data for these three peptides are summarized in Table 1. CCZ01096 was prepared in high purity and the identity was verified by mass spectrometry. *In vitro* competition binding assays showed that all three peptides had sub-nanomolar inhibition constants (K_i_: 0.31–0.59 nM) towards MC1R (Table 1).

**Table 1:** Analytical data for αMSH analogues and binding affinities for MC1R. a, data from^10^. b, data from^9^.

Peptide	Mass calculated	Mass found	Purity (%)	K_i_ (nM, n = 3)
^nat^Ga-CCZ01048^a^	1574.71	1576.12 (M + 2 H)	> 99%	0.31 ± 0.06
CCZ01064^b^	1369.73	1371.90 (M + 2 H)	> 97%	0.59 ± 0.05
CCZ01096	1745.93	1746.92 (M + 1 H)	> 99%	0.51 ± 0.08

### Radiochemistry and *in vivo* stability

The radiochemistry data for the preparation of [^68^Ga]Ga-CC01048, [^18^F]CCZ01064 and [^18^F]CCZ01096 are summarized in Table 2. ^68^Ga-labeling of CCZ01048 was achieved with high radiochemical yield (52.5 ± 3.7%) and high molar activity (315 ± 146 MBq/nmol). In contrast, a lower radiochemical yield (14.3 ± 4.9%) and molar activity (78.6 ± 21.0 MBq/nmol) was observed for ^18^F-labeled CCZ01064. To increase the molar activity of the ^18^F-labeled αMSH analogue, CCZ01096 with two AmBF_3_ motifs was designed and synthesized. ^18^F-Labeling of CCZ01096 resulted in a much higher radiochemical yield (29.2 ± 4.6%) and molar activity (193 ± 73.5 MBq/nmol) compared to [^18^F]CCZ01064. *In vivo* plasma stability of [^18^F]CCZ01096 was determined to be 79.2 ± 1.9% at 15 min p.i. using radio-HPLC (n = 3, Supplementary Fig. S3).

**Table 2:** Radiochemistry data for ^68^Ga-labeled CCZ01048, and ^18^F-labeled CCZ01064 and CCZ01096 (n ≥ 3).

Peptide	Radiochemical yield	Radiochemical purity (%)	Molar activity (MBq/nmol)
[^68^Ga]Ga-CCZ01048	52.5 ± 3.7	≥ 95	315 ± 146
[^18^F]CCZ01064	14.3 ± 4.9	≥ 95	78.6 ± 21.0
[^18^F]CCZ01096	29.2 ± 4.6	≥ 95	193 ± 73.5

### μPET imaging and biodistribution with [^18^F]CCZ01064

**Figure 3:**
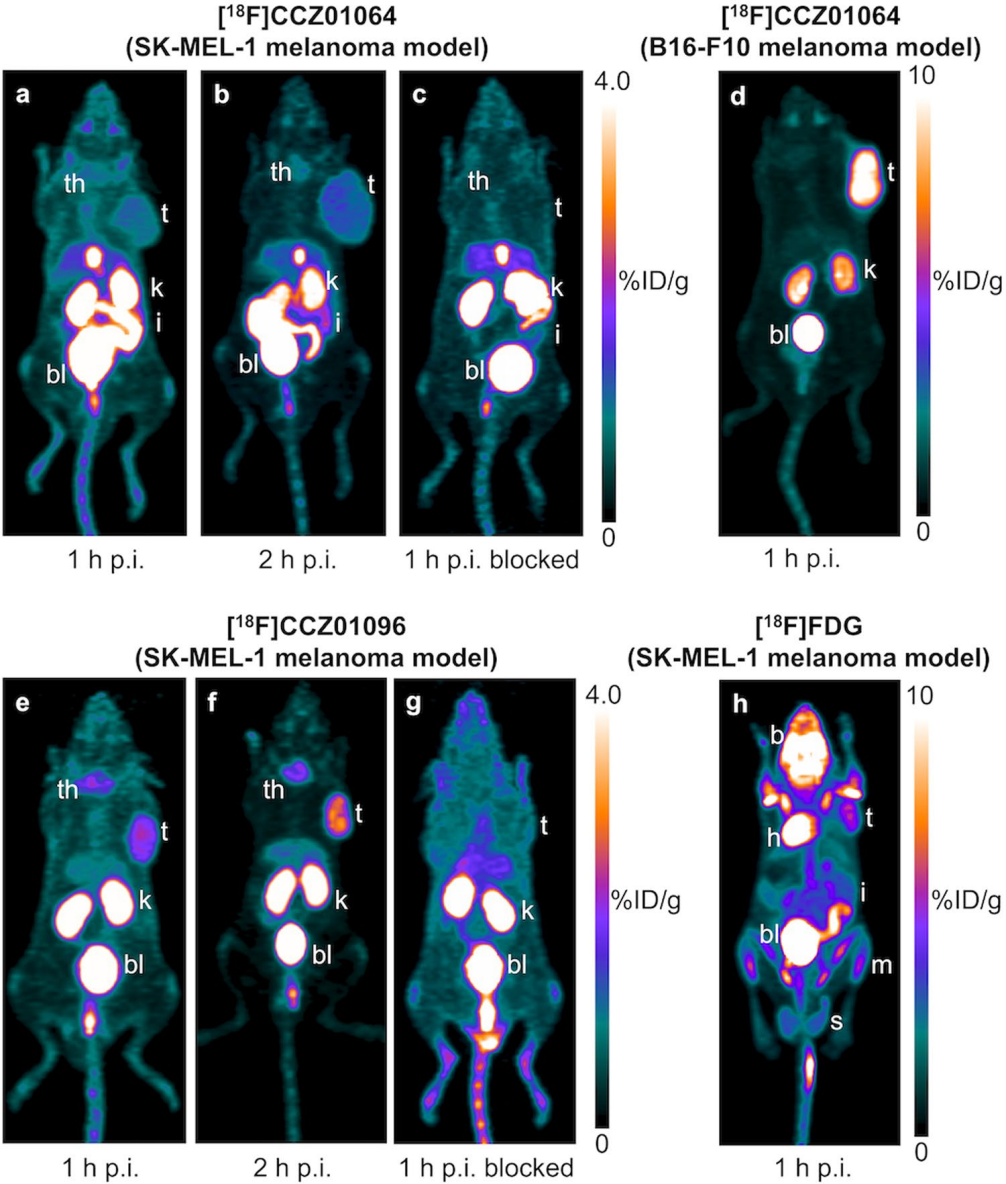
Representative μPET images of [^18^F]CCZ01064 at (**a)** 1 h p.i., (**b)** 2 h p.i., (**c)** 1 h p.i. blocked with co-injection of excess amount of non-radioactive CCZ01064 in NSG mice bearing human SK-MEL-1 melanoma xenografts, and (**d**) 1 h p.i. in C57BL/6 J mice bearing mouse B16-F10 melanoma. μPET images of [^18^F]CCZ01096 at (**e)** 1 h p.i., (**f)** 2 h p.i., and (**g)** 1 h p.i. blocked with co-injection of excess amount of non-radioactive CCZ01096 in NSG mice bearing human SK-MEL-1 melanoma xenografts. (**h**) μPET image of [^18^F]FDG at 1 h p.i. in NSG mice bearing human SK-MEL-1 melanoma xenografts. t, tumor; th, thyroid; k, kidney; bl, bladder; i, intestines; b, brain; h, heart; m, muscle; s, seminal glands.

μPET imaging was performed in male immunodeficient NOD.Cg-Prkdc^scid^ Il2rg^tm1Wjl^/SzJ (NSG) mice bearing human SK-MEL-1 tumor xenografts at 1 and 2 h p.i. for unblocked experiments, and at 1 h p.i. for blocked experiments with co-injection of excess amount (≥100 pmol) of non-radioactive CCZ01064 (Fig. 3a–c). Moderate tumor uptake and image contrast were observed on μPET images. Nonetheless, the tumors were clearly visualized at 2 h p.i. with radioactivity substantially cleared from normal tissues when compared to the image acquired at 1 h p.i. Co-injection with excess amount of non-radioactive CCZ01064 completely abolished the tumor uptake, suggesting the uptake in SK-MEL-1 tumor xenograft is receptor mediated. μPET imaging was also performed in male C57BL/6J mice bearing mouse B16-F10 melanoma at 1 h p.i. (Fig. 3d). As observed previously, very high tumor uptake and excellent tumor-to-background contrast was acquired.

**Figure 4:**
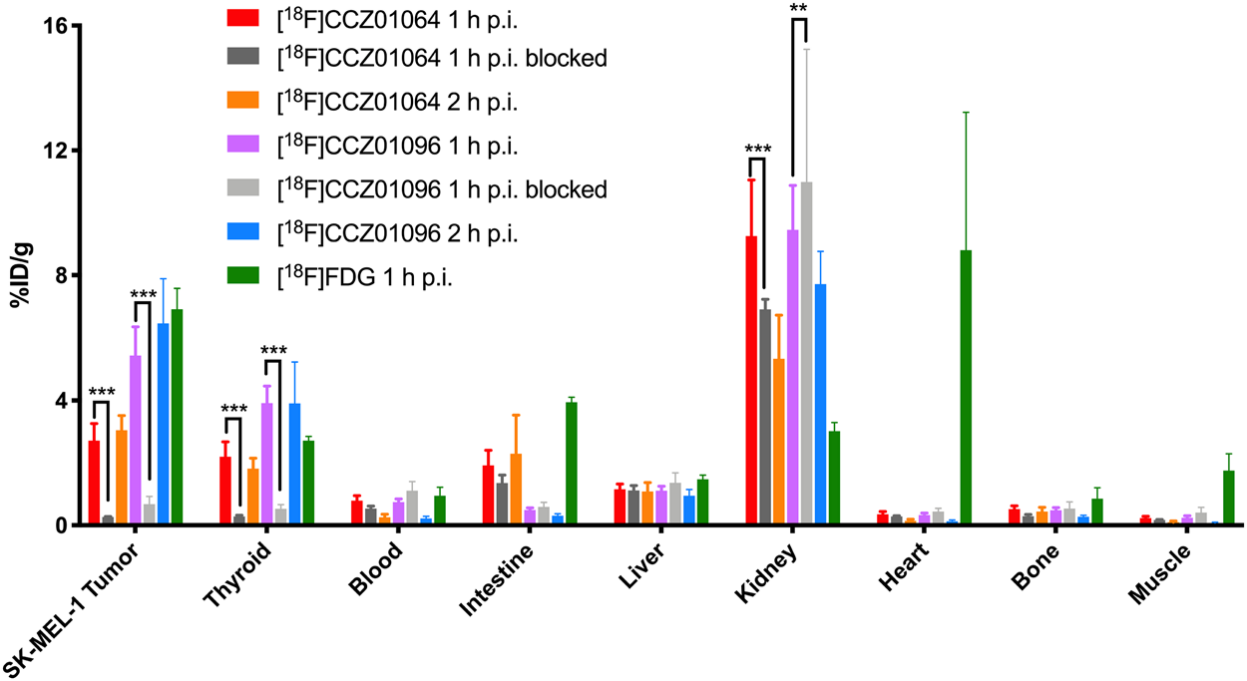
The biodistribution data of ^18^F-labeled CCZ01064 and CCZ01096 as well as [^18^F]FDG in SK-MEL-1 human melanoma-bearing mice. Multiple *t* tests were used to compare 1 h p.i. vs 1 h p.i. blocked, multiple comparisons were corrected using the Holm-Sidak method, **p < 0.01, ***p < 0.001, n = 5. Blocking studies were performed by co-injection of excess amount of non-radioactive CCZ01064 or CCZ01096 (≥100 pmol).

The biodistribution data were consistent with the results obtained from μPET imaging. The tumor uptake of [^18^F]CCZ01064 was 2.71 ± 0.55 and 3.05 ± 0.47%ID/g at 1 and 2 h p.i., respectively (Fig. 4, Supplementary Table S1). Minimal radioactivity accumulation was observed for most background tissues except thyroid, kidneys and intestines. At 2 h p.i., the tumor-to-blood and tumor-to-muscle ratios were 13.8 ± 6.2 and 33.7 ± 14.9, respectively. For the unblocked biodistribution studies, the injected radioactivity of [^18^F]CCZ01064 was 1.05 ± 0.29 MBq, corresponding to peptide mass of 21.62 ± 5.09 pmol (n = 10). With co-injection of non-radioactive CCZ01064 (≥100 pmol), the tumor uptake was significantly reduced to 0.27 ± 0.02%ID/g (90% reduction) at 1 h p.i.

### Effect of molar activity on tumor uptake

**Figure 5:**
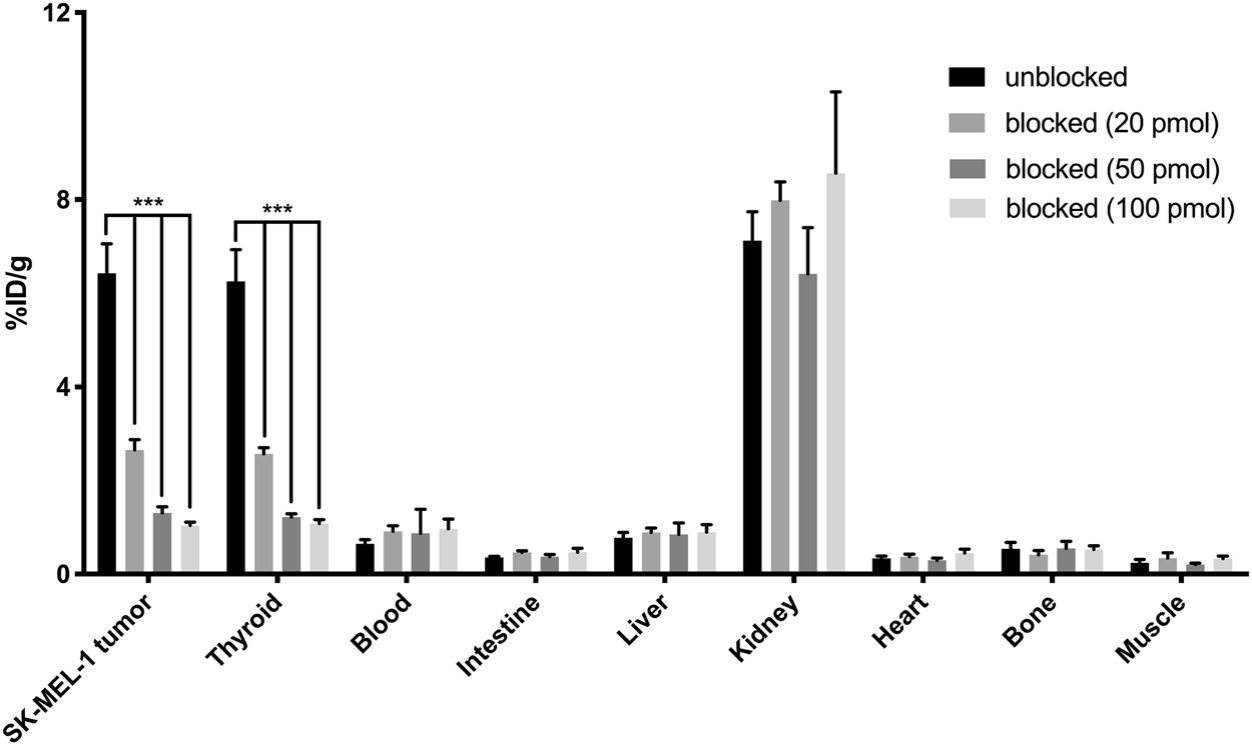
Biodistribution data of [^68^Ga]Ga-CCZ01048 at 1 h p.i. in NSG mice bearing human SK-MEL-1 tumor without and with co-injection of 20, 50 and 100 pmol of ^nat^Ga-CCZ01048 (Two-way ANOVA, multiple comparisons corrected using the Holm-Sidak method, ***p < 0.001, n = 4).

Biodistribution studies with [^68^Ga]Ga-CCZ01048 (1.38 ± 0.22 MBq) using NSG mice bearing human SK-MEL-1 tumor xenografts was performed at 1 h p.i. without or with co-injection of ^nat^Ga-CCZ01048 (Fig. 5). Without co-injection, the total injected peptide mass was 5.9 ± 0.90 pmol, which resulted in a tumor uptake of 6.15 ± 0.22%ID/g (n = 4). Co-injection of 20, 50 and 100 pmol of ^nat^Ga-CCZ01048 resulted in total injected peptide masses of 27.5 ± 0.6, 57.3 ± 1.0 and 109 ± 1.0 pmol (n = 4), respectively, and the corresponding tumor uptake values were reduced to 2.66 ± 0.22%ID/g (57% reduction), 1.31 ± 0.13%ID/g (79% reduction) and 1.04 ± 0.17%ID/g (83% reduction), respectively. Thyroid uptake showed a similar trend in reduction, but not other normal tissues (Fig. 5). This suggests that molar activity plays an important role on tumor uptake of human SK-MEL-1 melanoma xenograft which has a relatively low MC1R expression level.

### Design, μPET imaging and biodistribution of [^18^F]CCZ01096

In order to improve the molar activity of [^18^F]CCZ01064, CCZ01096 was designed to incorporate two AmBF_3_ motifs at both the α- and ε-amino groups of a lysine residue (Fig. 1). CCZ01096 showed sub-nanomolar inhibition constant for MC1R (K_i_ = 0.51 ± 0.08, n = 3, Table 1 and Supplementary Fig. S4), which was comparable to that of CCZ01064, suggesting adding one more AmBF_3_ motif did not affect the MC1R binding affinity. For radiolabeling of CCZ01096 with ^18^F, 104% and 146% increase in average radiochemical yield and molar activity was achieved, respectively, compared to those of [^18^F]CCZ01064 (Table 2).

μPET imaging with [^18^F]CCZ01096 in NSG mice bearing human SK-MEL-1 tumor xenografts showed significantly improved tumor-to-background contrast compared to [^18^F]CCZ01064, and the tumors were clearly visualized at both 1 and 2 h p.i. (Fig. 3e–f). Minimal radioactivity accumulation was observed at 2 h p.i. in most background tissues except thyroid and kidneys, and higher tumor-to-background contrast was also achieved at this time point. Tumor uptake was abolished by co-injection of excess amount of non-radioactive CCZ01096 (Fig. 3g).

Biodistribution data showed ~1-fold increase in tumor uptake with [^18^F]CCZ01096 (Fig. 4, Supplementary Table S1; 1 h p.i: 2.71 ± 0.55 vs. 5.44 ± 0.90%ID/g; 2 h p.i.: 3.05 ± 0.47 vs. 6.46 ± 1.42%ID/g) compared to data obtained with [^18^F]CCZ01064. Minimal radioactivity accumulation was observed in most background tissues except thyroid (3.92 ± 0.55 and 3.91 ± 1.32%ID/g at 1 and 2 h p.i., respectively) and kidneys (9.45 ± 1.42 and 7.72 ± 1.05%ID/g at 1 and 2 h p.i., respectively). Excellent tumor-to-background contrast (tumor-to-blood ratio at 30.6 ± 5.7 and tumor-to-muscle ratio at 85.7 ± 11.3) was achieved at 2 h p.i. For the unblocked biodistribution studies, the injected radioactivity of [^18^F]CCZ01096 was 0.72 ± 0.12 MBq, corresponding to a peptide mass of 4.20 ± 0.87 pmol (n = 10). With co-injection of excess amount of non-radioactive CCZ01096 (≥100 pmol), tumor uptake significantly decreased to 0.68 ± 0.25%ID/g (88% reduction).

### μPET imaging and biodistribution of [^18^F]FDG

μPET imaging and biodistribution studies using [^18^F]FDG in mice bearing the human SK-MEL-1 tumors were performed. SK-MEL-1 tumors were clearly visualized using [^18^F]FDG at 1 h p.i. (Fig. 3h). High radioactivity accumulation was also observed in the brain, heart, intestines, seminal glands and part of the leg muscle, with low bone uptake. Biodistribution showed consistent results, and the radioactivity uptake values for SK-MEL-1 tumor, brain, heart, intestines, seminal glands, muscles and bones were 6.91 ± 0.66, 9.54 ± 1.69, 8.80 ± 4.42, 3.95 ± 0.16, 4.63 ± 0.57, 1.75 ± 0.54 and 0.86 ± 0.35%ID/g at 1 h p.i., respectively (Fig. 4, Supplementary Table S1).

## Discussion

MC1R-targeted imaging of melanoma has been extensively studied using mouse melanoma models, most notably, the B16-F10 mouse melanoma cell line which has an exceptionally high receptor density at an average of 22,000 copies of per cell^24^. The use of mouse melanoma models facilitated the development of a large number of αMSH derivatives for μPET and μSPECT imaging^17^. However, the reported tumor uptake (<3.30%ID/g) and imaging contrast were sub-optimal when applying the αMSH-derived SPECT tracers for imaging human melanoma xenografts. For instance, tumor uptake values of 3.26 ± 1.23%ID/g (^99m^Tc(EDDA)-HYNIC-AocNle-CycMSH_hex_)^19^, 2.69 ± 0.78%ID/g (^99m^Tc-RGD-Lys-(Arg^11^)CCMSH), 1.08 ± 0.47%ID/g (^99m^Tc-RAD-Lys-(Arg^11^)CCMSH), 1.20 ± 0.72%ID/g (^99m^Tc-RGD-Lys-(Arg^11^)CCMSHscramble)^22^, 2.35 ± 0.12%ID/g (^99m^Tc-RGD-Aoc-(Arg^11^)CCMSH), and 1.71 ± 0.25%ID/g (^99m^Tc-RGD-PEG_2_-(Arg^11^)CCMSH)^20^ at 2 h p.i. were reported using M21 human melanoma-bearing mice; and 1.98 ± 0.26%ID/g (^188^Re-CCMSH) and 3.06 ± 0.68%ID/g (^188^Re-(Arg^11^)CCMSH) at 1 h p.i. were reported using TXM13 human melanoma bearing mice^21^. ^18^F-labeled αMSH radioligands have yet to be developed and tested for human melanoma imaging with μPET. In this study, we employed both ^18^F and ^68^Ga PET isotopes, with ^18^F being the focus, due to its higher positron emission (97% vs 88%), longer half-life (109.8 vs 67.7 min), and the fact that ^18^F could be produced in large quantities at TBq level via a medical cyclotron compared to ^68^Ga at GBq level via a ^68^Ge/^68^Ga generator.

The difficulty in using established human melanoma cell lines as preclinical tumor models lies in their much lower MC1R density. The reported MC1R density in human M21 melanoma cells is only 1,281 copies per cell^22^, which is approximately 16 times less than that in B16-F10 mouse melanoma cells. Since the M21 cell line was not commercially available at the time of the study, the SK-MEL-1 cell line was chosen as a human melanoma xenograft model based on high MC1R mRNA and DNA copy numbers reported in CCLE. MC1R protein expression in SK-MEL-1 was confirmed by Western Blotting, and the expression level was subsequently quantified by saturation binding assays to be 972 ± 154 copies per cell, which is slightly lower than the M21 cell line. The low MC1R expression level presents significant challenges for molecular imaging of human melanoma compared to other cancer imaging markers such as the prostate-specific membrane antigen (PSMA, > 250,000 receptors/cell on the LNCaP human prostate cancer cell line)^25^, the somatostatin-2a receptor (Sst2a, >28,000 receptors/cell on the ZR-75-1 human breast cancer cell line)^26^ or various integrin receptors (e.g. integrin α_v_β_3_, >120,000 receptors/cell on the U87MG human glioma cell line)^27^. This challenge was illustrated by using the same radioligand [^18^F]CCZ01064 to image mouse B16-F10 melanoma (22,000 MC1R/cell, Fig. 3d), compared to the human SK-MEL-1 melanoma xenograft (<1,000 MC1R/cell, Fig. 3a) at the same time point.

[^18^F]CCZ01064 generated moderate tumor uptake (3.05 ± 0.47%ID/g) and image contrast in mice bearing SK-MEL-1 tumor xenografts. Compared to most of ^68^Ga-labeled tracers prepared in our lab, ^18^F labeling via ^18^F-^19^F isotope exchange reaction yields ^18^F-labeled products with relatively low molar activity. As shown in Table 2, the average molar activities for [^68^Ga]Ga-CCZ01048 and [^18^F]CCZ01064 were 315 and 78.6 MBq/nmol, respectively. This is because [^68^Ga]Ga-CCZ01048 was purified by high-performance liquid chromatography (HPLC), and the unlabeled CCZ01048 was removed to achieve a higher molar activity. In contrast, ^18^F-labeled CCZ01064 and unlabeled ^19^F-CCZ01064 could not be separated using HPLC, thus a lower molar activity was observed. We explored the effect of molar activity on tumor uptake using [^68^Ga]Ga-CCZ01048 as it can be prepared with a much higher molar activity. Without co-injection of non-radioactive CCZ01048, the total injected mass of [^68^Ga]Ga-CCZ01048 was 5.1 ± 0.9 pmol, and the tumor uptake value at 1 h p.i. was 6.15 ± 0.22%ID/g. Co-injection of 20 pmol of ^nat^Ga-CCZ01048 resulted in a 57% reduction in tumor uptake, indicating the low MC1R expression in human melanoma led to receptor saturation even with only low pmol of injected MC1R-targeting peptides. Therefore, high molar activity of radiotracers is essential to achieve high tumor uptake and high-contrast μPET images in the preclinical human melanoma model used in this study.

To augment the molar activity, we designed a new analogue, CCZ01096, which had similar MC1R binding affinity compared to CCZ01064, and incorporated two AmBF_3_ motifs for radiolabeling. ^18^F-radiolabeling was performed via a simple one-step ^18^F-^19^F isotope exchange reaction in mild aqueous condition (pH 2.0) at 85 °C for 20 min. Due to a higher concentration of the AmBF_3_ motif available for CCZ01096 at a given overall concentration of precursor, a higher radiochemical yield was obtained, leading to a higher molar activity (Table 2). The higher molar activity allowed lower peptide mass to be injected, i.e. 4.20 ± 0.87 pmol for [^18^F]CCZ01096 vs 21.62 ± 5.09 pmol for [^18^F]CCZ01064 in the biodistribution studies. The lower injected mass of [^18^F]CCZ01096 also resulted in improved tumor uptake (6.46 ± 1.42%ID/g, 2 h p.i.) and image contrast (tumor-to-blood: 30.6 ± 5.71; tumor-to-muscle: 85.7 ± 11.3) compared to those of [^18^F]CCZ01064 (Supplementary Table S1).

For ^18^F-labeled CCZ01064 and CCZ01096, low bone uptake was observed from μPET images and biodistribution data, suggesting that minimal defluorination occurred for both radiotracers. In addition, [^18^F]CCZ01096 was considerably stable *in vivo* with 79.2 ± 1.9% remaining intact in plasma at 15 min p.i. Moreover, co-injection of excess amount of nonradioactive MC1R-taregting peptides abolished uptake of both radiotracers into tumor xenografts, suggesting radioactivity accumulation in SK-MEL-1 tumor xenografts was MC1R mediated. Delayed radioactivity clearance was observed in the blocked studies due to the higher amount of injected peptide mass, which resulted in overall higher background radioactivity level. The same phenomenon was also observed in our previous study using [^18^F]CCZ01064 and mice bearing the B16-F10 mouse melanoma model^9^. Interestingly, moderate thyroid uptake was also observed, and the thyroid uptake level was significantly decreased in mice co-injected with excess amount of non-radioactive MC1R-targeting peptides (Fig. 4, Supplementary Table S1), suggesting potential off-target binding of both radiotracers, or the presence of melanocortin receptors in the thyroid gland.

To compare with the current “gold standard” in the clinic, μPET imaging and biodistribution studies using [^18^F]FDG in mice bearing the human SK-MEL-1 tumors were also performed. The [^18^F]FDG used in the study was clinical grade, as it was collected as an aliquot from the BC Cancer clinic, which was used for human patient scans. SK-MEL-1 tumor uptake with [^18^F]FDG showed slightly higher values at 6.91 ± 0.66%ID/g compared to 5.44 ± 0.90%ID/g for [^18^F]CCZ01096 at 1 h p.i. However, overall high background tissue uptake was observed, particularly in the brain, heart, intestines and seminal glands. This is expected, as the high metabolic rate of cells led to the high uptake. Also, moderate radioactivity accumulation was observed in the leg muscles, due to the fact that following tail vein radiotracer injection, mice were allowed to roam in their cages until the 1 h p.i. time point. Low bone uptake indicated minimal *in vivo* defluorination. These results underscored the high specificity of the αMSH derivatives, which produced high tumor-to-background contrast images.

In conclusion, we evaluated two ^18^F-labeled αMSH derivatives for preclinical μPET imaging of human melanoma. The SK-MEL-1 cell line with ~972 copies of MC1R/cell was successfully used as a human melanoma model for tracer evaluation. Due to a lower MC1R expression level, a higher molar activity of the radiotracers is required for preclinical imaging using human melanoma models. [^18^F]CCZ01096 with two AmBF_3_ motifs to achieve higher radiochemical yield and molar activity was successfully used to visualize human SK-MEL-1 melanoma xenografts in μPET images. With higher tumor uptake and tumor-to-background contrast, [^18^F]CCZ01096 warrants further evaluation for potential clinical translation to detect metastatic melanoma.

## Methods

### Cell culture

The B16-F10 mouse melanoma (CRL-6475) and SK-MEL-1 human melanoma (HTB-67) cell lines were obtained from the American Type Culture Collection. The cell lines were confirmed pathogen-free by the IMPACT 1 mouse profile test (IDEXX BioResearch). The B16-F10 and SK-MEL-1 cells were cultured in Dulbecco’s Modified Eagle’s Medium and Eagle’s Minimum Essential Medium (StemCell Technologies), respectively, at 37 °C in a humidified incubator containing 5% CO_2_. Both media were supplemented by 10% FBS, 100 U/mL penicillin and 100 μg/mL streptomycin.

### Receptor saturation assays

MC1R receptor density on SK-MEL-1 cells was evaluated using saturation binding assays according to previously published procedures^16^. Briefly, approximately 450,000 SK-MEL-1 cells/well were added to 96 well V-bottom plates in reaction buffer (Roswell Park Memorial Institute media, 4.8 mg/mL HEPES, 2 mg/mL BSA, 100 U/mL penicillin and 100 μg/mL streptomycin). Increasing concentrations (20 pM to 2 nM) of [^125^I][Nle^4^, D-Phe^7^]-αMSH ([^125^I]NDP-αMSH, PerkinElmer) was added to the cells and incubated for 1 h at 25 °C with mild agitation. Non-specific binding was determined by co-incubation with non-radioactive NDP-αMSH (10 μM). The cells were washed with ice-cold phosphate buffered saline (PBS) twice, and measured for radioactivity on a WIZARD 2480 gamma counter (PerkinElmer).

### Peptide synthesis

Peptides were synthesized on solid phase using Fmoc chemistry according to previously published procedures for CCZ01048^10^ and CCZ01064^9^. For CCZ01096, Fmoc-Pip-Nle-CycMSH_hex_ was synthesized on Rink-Amide-MBHA resin as described previously^9^. To obtain the two AmBF_3_ motifs, the Fmoc protection group was removed with 20% piperidine in dimethylformamide (DMF), followed by coupling of Fmoc-Lys(Fmoc)-OH (3 eq.) in the presence of HBTU (3 eq.), HOBt (3 eq.) and DIEA (6 eq.). The Fmoc protection groups on the N^α^ and lysine side chain were simultaneously removed, followed by coupling of 2-azidoacetic acid (10 eq.) in the presence of *N*,*N*′-diisopropylcarbodiimide (10 eq.) and *N*-hydroxysuccinimide (12 eq.). The azide-containing peptide was cleaved from the resin by treating with 92.5/2.5/2.5/2.5/ trifluoroacetic acid (TFA)/Phenol/H_2_O/triisopropylsilane for 3 h at room temperature. The solution was filtered and the peptides in filtrate were precipitated by treating with diethyl ether. After filtration, the desired peptide was purified using HPLC (Agilent) with a semi-preparative C18 column (Phenomenex Luna, 5 μm, 250 × 10 mm). The mass of the diazide-containing peptide was verified by mass spectrometry (AB/Sciex 5600). Mass calculated [M + 2 H]^2+^ 708.87, found 708.82 (Supplementary Fig. S5).

The dual AmBF_3_-containing CCZ01096 was synthesized via click reaction by incubating the purified diazide-containing peptide with *N*-propargyl-*N*,*N*-dimethyl-ammoniomethyl-trifluoroborate (10 eq.), CuSO_4_ (10 eq.), sodium ascorbate (24 eq.) in 80/20 H_2_O and acetonitrile at 45 °C for 2 h. The mixture was purified by HPLC with 29% acetonitrile and 71% ammonium formate (40 mM, pH 6.0) at a flow rate of 4.5 mL/min. The purity was determined using HPLC with an analytical C18 column (Phenomenex Luna, 5 μm, 250 × 4.6 mm). Mass calculated [M + 1 H]^+^ 1746.93, found 1746.92 (Supplementary Fig. S6).

### Receptor binding assays

Receptor binding affinity of CCZ01096 was assessed using previously published procedures^10^. Briefly, approximately 500,000 B16-F10 cells/well were seeded onto 24 well poly-D-lysine plates overnight. The cells were washed with PBS and the growth media was replaced by the reaction buffer described above. Increasing concentrations of non-radioactive CCZ01096 (0.5 pM to 5 μM) along with 0.1 nM of [^125^I]NDP-αMSH were also added. The reaction mixture was incubated at 25 °C with moderate agitation for 1 h. Cells were washed with ice-cold PBS, harvested, and measured for radioactivity on a WIZARD 2480 gamma counter.

### Radiochemistry

The ^68^Ga labeling of CCZ01048 and ^18^F labeling of CCZ01064 were performed according to previously published procedures^9,10^. The HPLC purification condition for [^68^Ga]Ga-CCZ01048 was 21% acetonitrile containing 0.1% TFA at a flow rate of 4.5 mL/min using a semi-preparative C18 column, the retention time was 24.4 min. The retention time for the CCZ01048 precursor was 21.3 min using the same condition. For [^18^F]CCZ01064, 30% acetonitrile and 70% NH_4_HCO_2_ (40 mM, pH 6.0) was used at a flow rate of 4.5 mL/min, and the retention time was 17.7 min.

For radiolabeling of CCZ01096 with ^18^F, ^18^F-fluoride was produced via a TR19 cyclotron (Advanced Cyclotron Systems Inc.). Approximately 37–74 GBq of ^18^F-fluoride was mixed with ^19^F-CCZ01096 (100 nmol) in 1:1 (v/v) solution of pyridazine-HCl buffer (1 M, pH 2.0) and DMF. The mixture was incubated at 85 °C for 20 min, quenched with PBS and purified by HPLC with 29% acetonitrile and 71% ammonium formate (40 mM, pH 6.0) at a flow rate of 4.5 mL/min, the retention time for [^18^F]CCZ01096 was 20.5 min. The collected HPLC eluate was diluted with water (50 mL) and the mixture was passed though a C18 Sep-Pak cartridges. The radioactivity trapped on the cartridge was eluted off with ethanol (0.5 mL) and formulated in saline for μPET imaging and biodistribution studies. Quality control was performed via HPLC using an analytical C18 column, and co-elution of [^18^F]CCZ01096 with the non-radioactive counterpart was used to confirm the identity of the ^18^F-labeled product. Molar activity was calculated at the end of synthesis by dividing collected radioactivity by mass of the peptide, which was determined by a previously prepared standard curve (UV peak area vs injected mass) using HPLC with UV absorption at λ = 220 nm. [^18^F]FDG was acquired as an aliquot from the clinical production site at BC Cancer (Batch# FDG20190807A).

### Tumor implantation

All animal experiments were conducted in according to the guidelines established by Canadian Council on Animal Care and approved by Animal Ethics Committee of the University of British Columbia. Male C57BL/6 J and immunodeficient NSG mice were obtained from an in-house breeding colony at the BC Cancer Research Centre. For tumor implantation, mice were anesthetized by inhalation with 2% isoflurane, 1 × 10^6^ B16-F10 and 5 × 10^6^ SK-MEL-1 cells were inoculated subcutaneously on the right dorsal flank of C57BL/6J and NSG mice, respectively. Mice were used for imaging or biodistribution studies when the tumors reached 6–8 mm in diameter.

### Preclinical μPET/μCT imaging and biodistribution

μPET/μCT imaging and biodistribution experiments were performed on a μPET/μCT scanner (Inveon, Siemens) using previously published procedures^10^. Briefly, tumor-bearing mice were injected with approximately 3.0–5.0 MBq (for imaging) or <1.5 MBq (for biodistribution) of radiolabeled peptides via tail vein under sedation (2% isoflurane inhalation). For [^18^F]FDG imaging and biodistribution, mice underwent fasting overnight prior to the experiments. Imaging studies were performed in duplicate or triplicate. For biodistribution, the injected mass was 21.62 ± 5.09 pmol (n = 10) for [^18^F]CCZ01064 and 4.20 ± 0.87 pmol (n = 10) for [^18^F]CCZ01096. For blocking studies, radiolabeled peptides were co-injected with excess amount of non-radioactive counterparts (≥100 pmol). In the molar activity testing experiment for [^68^Ga]Ga-CCZ01048, the radiotracer was co-injected with 0, 20, 50 and 100 pmol of non-radioactive ^nat^Ga-CCZ01048 (n = 4). After injection, mice were allowed to recover and roam freely in their cages. At 1 or 2 h p.i., mice were anesthetized again and positioned in the scanner. A baseline CT scan was obtained for localization and attenuation correction, followed by a 10–15 min static μPET scan. For biodistribution studies, mice were euthanized by CO_2_ inhalation, blood was withdrawn promptly, and the tissues of interest were harvested, rinsed, and blotted dry. All tissues were weighed and the radioactivity of the collected samples was measured using a WIZARD 2480 gamma counter.

### Statistical analysis

Statistical analysis was performed using GraphPad Prism 7.0a. Multiple *t*-tests were performed for comparing unblocked and blocked groups in the biodistribution studies. Two-way ANOVA was performed to test which group of organs was affected by increasing amount of the blocking agent in the [^68^Ga]Ga-CCZ01048 biodistribution studies. Multiple comparisons were corrected using the Holm-Sidak method. Outliers were removed using the ROUT method with Q = 1%. The difference was considered statistically significant when *p* value was < 0.05.

## Supplementary information

Supplementary Information
