# Supplementary material for: 18F-Labeled Cyclized α-Melanocyte-Stimulating Hormone Derivatives for Imaging Human Melanoma Xenograft with Positron Emission Tomography
Source: Sci Rep. 2019 Sep 19;9:13575. doi: 10.1038/s41598-019-50014-5 (PMC6753210; doi:10.1038/s41598-019-50014-5)
Supplement: Supplementary file 1 — Supplementary Information [file 41598_2019_50014_MOESM1_ESM.docx]

**Supplementary Information for Manuscript entitled**

“**^18^F-Labeled Cyclized α-Melanocyte-Stimulating Hormone Derivatives for Imaging Human Melanoma Xenograft with Positron Emission Tomography**”

Chengcheng Zhang^1^, Zhengxing Zhang^1^, Helen Merkens^1^, Jutta Zeisler^1^, Nadine Colpo^1^, Navjit Hundal-Jabal^1^, David M. Perrin^2^, Kuo-Shyan Lin*^1,3^,François Bénard*^1,3^

^1^Department of Molecular Oncology, BC Cancer, Vancouver, BC, Canada

^2^Department of Chemistry, and ^3^Department of Radiology, University of British Columbia, Vancouver, BC, Canada

**Supplementary Method**

**Western blotting**

SK-MEL-1 and MeWo cells were lysed in RIPA buffer (Thermofisher) plus protease inhibitors (cOmplete ULTRA tablets, Roche). Protein concentration was determined using the BCA method (Thermofisher). Approximately 10 μg of protein was loaded onto 8% SDS-PAGE gel. The proteins were transferred onto 0.45 µm nitrocellulose membrane (Amersham) using semi-dry transfer blotter (BioRad). The membrane was blocked with TBS-T buffer with 5% skim milk powder for 1 h and then incubated with the primary anti-MC1R antibody (1:1450, LS-C402431 LifeSpan Biosciences) overnight at 4 °C. Next the membrane was washed and then incubated with goat anti-rabbit-POD secondary antibody (1:2000, DAKO) for 2 h at room temperature. The blot was washed and developed using ECL Select (Amersham-GE Healthcare). The same blot was stripped with antibody stripping buffer (Gene Bio-Application) for 20 min, re-blocked and re-probed with anti-beta-actin (abcam ab119716). The images were acquired using an Image Quant LAS 4000 (GE Healthcare).

***In vivo* plasma stability**

*In vivo* plasma stability was performed using the same procedure as previously reported.^10^

**Supplementary Figure S1.** DNA copy number and mRNA expression data of MC1R among all the human melanoma cell lines archived in Cancer Cell Line Encyclopedia. SK-MEL-1 is showing high values in both categories, whereas MeWo shows lower values.

**Supplementary Figure S2.** Western blot results of (**a)** MC1R protein expression and (**b)** beta-actin control on SK-MEL-1 and MeWo human melanoma cell lines.

**
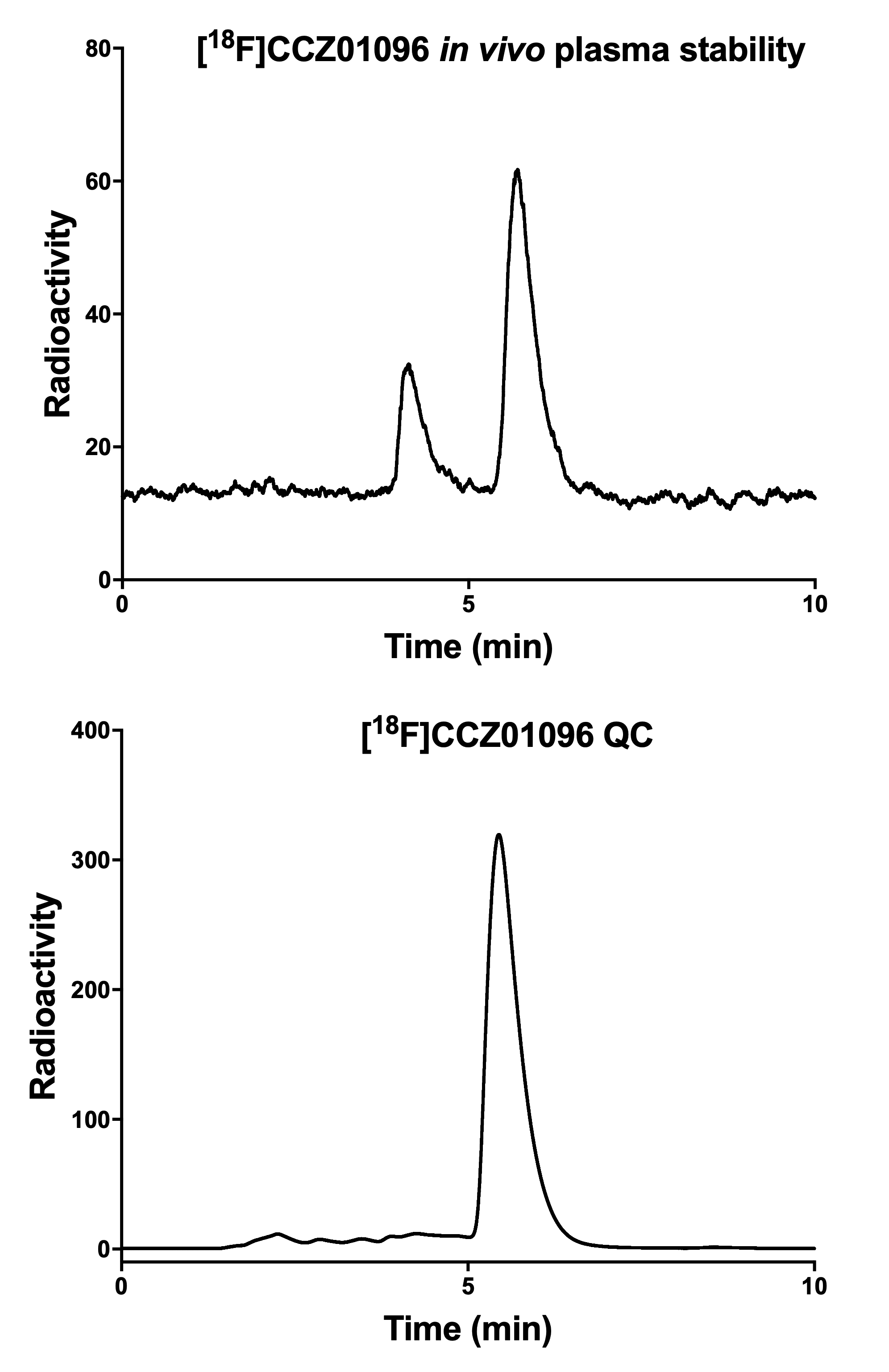
**

**Supplementary Figure S3.** Representative *in vivo* plasma stability results for [^18^F]CCZ01096 at 15 min p.i.

**
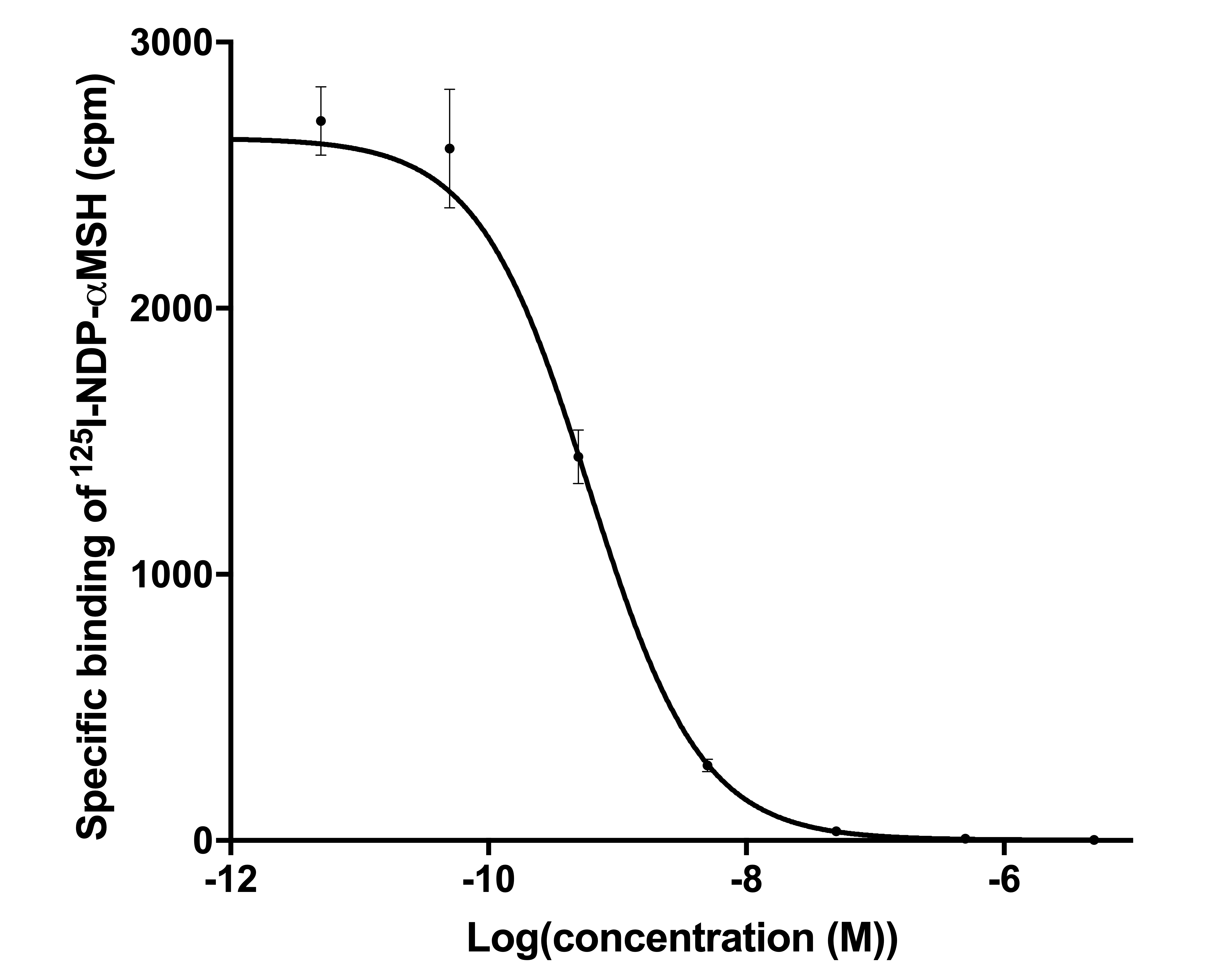
**

**Supplementary Figure S4.** Representative competitive binding curve for CCZ01096 on B16-F10 cells.

**
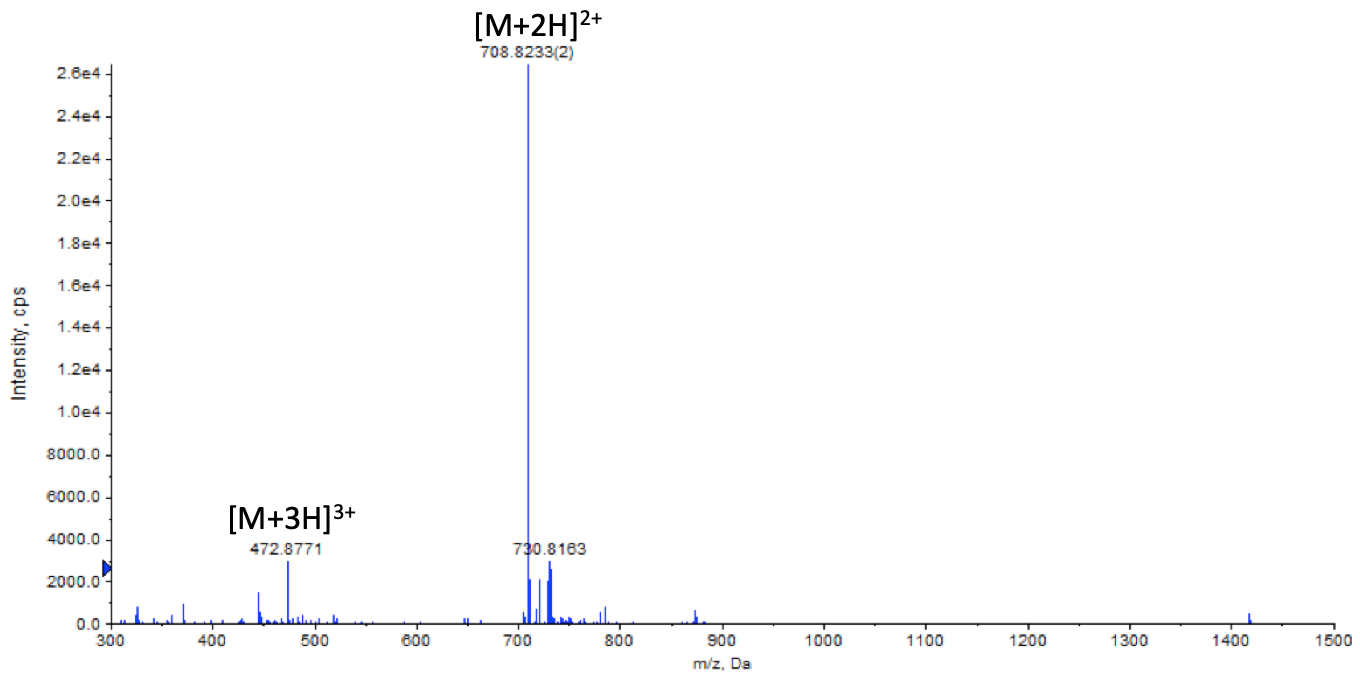
**

**Supplementary Figure S5.** Mass spectrometry result of the diazide-containing peptide precursor for CCZ01096. Mass calculated [M+2H]^2+^ 708.87, found 708.82.


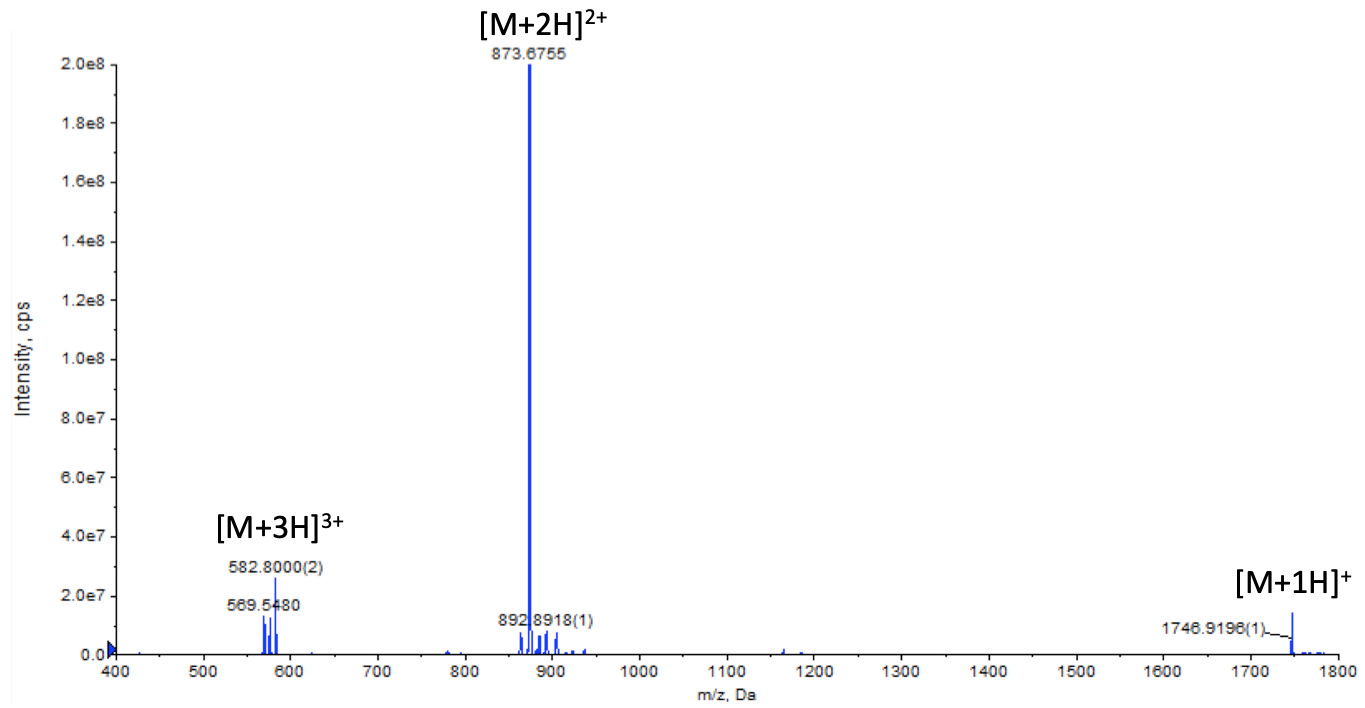


**Supplementary Figure S6.** Mass spectrometry result of CCZ01096. Mass calculated [M+1H]^+^ 1746.93, found 1746.92.

**Supplementary Table S1**. The biodistribution data of ^18^F-labeled CCZ01064 and CCZ01096, as wel as [^18^F]FDG in SK-MEL-1 human melanoma-bearing mice. Multiple *t* tests were used to compare 1 h p.i. vs 1 h p.i. blocked, multiple comparisons were corrected using the Holm-Sidak method, ** *p* < 0.01, *** *p* < 0.001, n = 5. Blocking studies were performed by co-injection of excess amount of non-radioactive CCZ01064 or CCZ01096 (≥ 100 pmol). ^a^n = 4.

| Tissue |  | | [^18^F]CCZ01064 | |  | [^18^F]CCZ01096 | | | [^18^F]FDG |  |
| --- | --- | --- | --- | --- | --- | --- | --- | --- | --- | --- |
|  | 1 h p.i. | 2 h p.i. | | 1 h p.i. blocked^a^ |  | 1 h p.i. | 2 h p.i. | 1 h p.i. blocked^a^ | 1 h p.i.^a^ | |
| SK-MEL-1 tumor | 2.71±0.55 | 3.05±0.47 | | 0.27±0.02*** |  | 5.44±0.90 | 6.46±1.42 | 0.68±0.25*** | 6.91±0.66 | |
| Blood | 0.79±0.15 | 0.26±0.10 | | 0.53±0.09 |  | 0.74±0.10 | 0.22±0.07 | 1.10±0.30 | 0.95±0.27 | |
| Fat | 0.17±0.10 | 0.19±0.15 | | 0.12±0.05 |  | 0.32±0.17 | 0.08±0.03 | 0.20±0.06 | 0.86±0.21 | |
| Seminal glands | 0.16±0.05 | 0.16±0.07 | | 0.23±0.24 |  | 0.22±0.03 | 0.12±0.05 | 0.15±0.04 | 4.63±0.57 | |
| Testes | 0.36±0.14 | 0.32±0.09 | | 0.22±0.03 |  | 0.41±0.15 | 0.23±0.03 | 0.42±0.13 | 1.15±1.03 | |
| Intestines | 1.92±0.48 | 2.29±1.24 | | 1.35±0.30 |  | 0.49±0.07 | 0.31±0.06 | 0.59±0.15 | 3.95±0.16 | |
| Spleen | 0.71±0.15 | 0.52±0.08 | | 0.48±0.07 |  | 0.80±0.22 | 0.48±0.03 | 0.87±0.29 | 3.59±0.68 | |
| Pancreas | 0.21±0.06 | 0.12±0.03 | | 0.15±0.02 |  | 0.25±0.07 | 0.08±0.01 | 0.28±0.07 | 1.41±0.20 | |
| Stomach | 0.19±0.07 | 0.14±0.05 | | 0.09±0.01 |  | 0.20±0.05 | 0.22±0.02 | 0.23±0.07 | 1.00±0.11 | |
| Liver | 1.15±0.17 | 1.08±0.29 | | 1.11±0.18 |  | 1.11±0.14 | 0.95±0.20 | 1.36±0.32 | 1.48±0.14 | |
| Adrenal glands | 0.82±0.31 | 0.61±0.31 | | 0.56±0.17 |  | 0.99±0.36 | 0.29±0.03 | 0.68±0.17 | 2.05±0.28 | |
| Kidneys | 9.26±1.80 | 5.34±1.38 | | 6.90±0.37*** |  | 9.45±1.42 | 7.72±1.05 | 11.0±4.25** | 3.01±0.28 | |
| Heart | 0.35±0.09 | 0.15±0.05 | | 0.28±0.02 |  | 0.33±0.07 | 0.13±0.04 | 0.44±0.11 | 8.80±4.42 | |
| Lungs | 1.26±0.22 | 0.83±0.33 | | 1.11±0.21 |  | 1.31±0.21 | 0.61±0.22 | 1.87±0.45 | 3.42±0.40 | |
| Thyroid | 2.20±0.48 | 1.82±0.33 | | 0.28±0.04*** |  | 3.92±0.55 | 3.91±1.32 | 0.53±0.14*** | 2.72±0.13 | |
| Bone | 0.52±0.11 | 0.44±0.13 | | 0.29±0.07 |  | 0.48±0.09 | 0.28±0.05 | 0.54±0.21 | 0.86±0.35 | |
| Muscle | 0.23±0.06 | 0.10±0.03 | | 0.17±0.03 |  | 0.24±0.06 | 0.08±0.02 | 0.41±0.17 | 1.75±0.54 | |
| Brain | 0.03±0.01 | 0.02±0.01 | | 0.02±0.00 |  | 0.02±0.00 | 0.02±0.01 | 0.03±0.01 | 9.54±1.69 | |
|  |  |  | |  |  |  |  |  |  | |
| Tumor/Blood | 3.43±0.46 | 13.8±6.19 | | 0.51±0.07*** |  | 7.51±1.79 | 30.6±5.71 | 0.60±0.07** | 7.64±2.06 | |
| Tumor/Muscle | 11.9±2.81 | 33.7±14.9 | | 1.61±0.25*** |  | 23.7±7.44 | 85.7±11.3 | 1.68±0.14*** | 4.22±1.30 | |
| Tumor/Bone | 5.27±1.12 | 7.63±3.22 | | 0.94±0.17*** |  | 11.6±2.71 | 23.5±3.52 | 1.28±0.17*** | 8.83±2.62 | |
| Tumor/Kidneys | 0.29±0.03 | 0.62±0.23 | | 0.04±0.00 |  | 0.58±0.12 | 0.83±0.12 | 0.06±0.02 | 2.30±0.04 | |
